# Health Belief and Behavioral Analysis of Fad Diets: A Perspective from Younger Generations in a Developing Country

**DOI:** 10.3390/foods13121858

**Published:** 2024-06-13

**Authors:** Ray Ver V. Baldemor, Ardvin Kester S. Ong, Josephine D. German, Netanya S. Bautista, Marc Lenard V. Alonso, Oldrin John P. Alidio

**Affiliations:** 1 School of Industrial Engineering and Engineering Management, Mapua University, 658 Muralla St., Intramuros, Manila 1002, Philippines; 2 E.T. Yuchengco School of Business, Mapua University, 1191 Pablo Ocampo Sr. Ext, Makati 1204, Philippines

**Keywords:** diet, fad diets, health motivation, health belief model, theory of planned behavior

## Abstract

The surge in popularity of fad diets has raised concerns about compromised health among individuals due to their beliefs and intentions regarding consumption. The aim of this study was to examine the prevalence of fad dieting among persons who are dieting and to determine the different factors influencing the inclination to adopt fad diets. Specifically, this study explored the ways in which individual openness to following fad diets, participation in diet trends, and characteristics may influence attitudes towards fad diet adoption. Data from 407 participants aged 18–34, collected via Google Forms, were analyzed using a high-ordered construct approach between the theory of planned behavior (TPB) and health belief model (HBM). Employing partial least squares structural equation modeling, significant results were obtained. The key findings revealed that knowledge about dieting, perceived benefits, and health motivation significantly influenced individuals’ intentions to adopt fad diets. Additionally, the study demonstrated significant impacts of health motivation on attitude and perceived behavioral control, subsequently affecting individuals’ intention to adopt dietary practices. Practical implications include the development of tailored health communication strategies for government agencies and informed decision-making support for individuals considering adopting fad diets. This research contributes valuable insights into the perception and psychological and social factors shaping dietary decisions, laying the groundwork for enhanced health education and intervention strategies. Furthermore, the study’s theoretical framework offers potential for extension and application to health-related food consumption behaviors across diverse cultural contexts.

## 1. Introduction

Popular diets that are frequently promoted as a quick remedy for weight loss are known as dietary fads (or fad diets). The claimed benefits of these diets often make them seem enticing, but the practices being promoted raise serious concerns [1]. As such, there is a growing recognition of the need for a critical evaluation of individual dietary practices, given the potential risks associated with fad diets. Moreover, with weight loss emerging as a major health concern globally [2], understanding the factors driving the adoption of these diets becomes paramount.

Leading worldwide nutrition brand Herbalife unveiled the results of the 2020 Diet Decisions Survey [3], showing that consumers in the Asia Pacific region are becoming more active and are trying to eat better, with 58% of them anticipating a healthier future under the new normal. According to the survey, 53% of Filipinos said they had started eating more fruits and vegetables, and 43% said they had started eating more plant-based meals. The Philippines also scored highest (62%) for being receptive to plant-based diets and vegetarian choices [4].

The National Nutrition Council (NCC) Governing Board of the Republic of the Philippines explained that fad diets are not encouraged for weight loss because they may pose health risks and dangers [5]. However, evident in the current generation, the keto diet, intermittent fasting, Atkins diet, blood type diet, and many more fad diets are being practiced around the world [6]; these diets are often characterized by nutritional imbalances, difficulty in adherence, and lack of sustainability, which can lead to organ stress, insufficient emphasis on physical activity, and potential psychological consequences. Research also suggests that women, particularly adolescents, are more inclined to adopt these fad diets [7,8].

Among the fad diets, the keto diet has become extremely popular in recent years [9]. Especially since many fitness enthusiasts and celebrities have shifted to the keto diet in an effort to lose weight, consumers have now considered this to be effective due to its advertisement and popularity. The keto diet works by fat breakdown as a source of energy instead of the common notion of utilizing carbohydrates [7]. Among its many claimed advantages are possible weight loss, acne control, and heart health enhancement [10]. With 25.4 million searches, “keto” was the most Googled food-related term worldwide in 2020. It was seen that previous fad diets like the Atkins diet and intermittent fasting have been surpassed by the keto diet [11].

On the other hand, vegan and vegetarian diets, as opposed to those that include meat and other animal products, are more environmentally friendly according to Springmann et al. [12]. It was pointed out that there are health and environmental advantages to replacing animal products with plant-based ones. According to the World Animal Foundation, around 1.5 billion people worldwide identify as vegetarians, accounting for 22% of the world’s population [13]. Presented in the study of Bali and Naik [14], this diet reduces the risk of cardiovascular diseases, obesity, and type II diabetes, and decreases liver diseases. However, the diet had several impacts such as negative mental effects, decrease in immune system function, and nutritional deficiencies.

Regarding different diet practices, a number of studies pointed to the detrimental effects on one’s health that result from following such eating regimens over time [15,16]. To add context, Prasad et al. [17] investigated the inter-relationships between body weight, body image, and the perceived desirability of fad diets among adolescent girls. It was discovered that the group that desired weight loss of more than ten percent regarded fad diets as being substantially more desirable than the group that desired weight loss of less than ten percent. Overweightness and obesity, together with their related factors and lifestyle changes, have become major health concerns in recent years, leading to a dramatic increase in the costs connected with medical treatment for individuals. Despite the availability of various treatments, fad diets continue to be advertised as the easiest and simplest approach to drop those extra pounds. Nonetheless, the adoption of a fad diet without proper medical guidance would lead to serious complications, especially since most people adopt fad diets depending on their perceptions, beliefs, and influences [14].

Contrarily, modifying one’s lifestyle, eating habits, and level of physical activity are the cornerstones of overweightness and obesity prevention and treatment. However, Wadden et al. [18] indicated that monitoring a person’s diet can significantly contribute to greater weight loss. While there is no doubt that fad diets can help people lose weight and reduce their risk of cardiovascular disease, research has shown that these programs can have serious consequences, including mortality in the long run [19]. In accordance, while fad diets may lead to short-term weight loss, there are long-term health implications associated with their sustained use. For example, Schaack [20] and Anderson et al. [21] explained that some fad diets involving severe calorie and nutrient restriction can result in fatigue, dehydration, and irregular bowel movements in the short term. While following fad diets can help one consume fewer calories overall each day, it also means that micronutrient deficiencies are prevalent [22]. The long-term effect of caloric and micronutrient deficiencies may even precipitate the development of disordered eating behaviors or irregular eating patterns [20,21].

Effective nutrition education interventions have the potential to influence individuals’ attitudes towards fad diets by addressing motivations [23,24], perceptions, and tailored learning experiences [25]. However, there remains a significant gap in understanding the psychological factors driving individuals’ decisions to adopt fad diets, highlighting the need for further research in this area. Specifically, there is still a significant knowledge gap about the complex variables driving people’s decisions to follow different fad diets. This raises the question of whether individuals are adopting fad diets because of their own knowledge and health beliefs or their own behavioral motivation.

Among related studies, the complex interactions between psychological, social, and cultural factors that influence people’s views and behaviors around fad diets have been generally ignored. The current body of research available tends to offer a broad perspective without exploring the many regional and cultural factors that influence people’s choices, which makes it more difficult to build focused treatments to encourage sustainable eating habits. Furthermore, while a number of studies discuss the impact of motivation and knowledge on dietary practices, there is a lack of research that thoroughly examines the influence of health belief factors, social norms, and behavior on people’s intentions regarding fad diets.

Consequently, a more comprehensive and context-specific investigation of the psychological elements impacting the propensity to follow fad diets is desperately needed. Closing this research gap would advance our knowledge of the intricacies involved in each person’s decision-making process when it comes to nutrition. With that, the aim of this study was to examine the prevalence of fad dieting among persons who are dieting and determine the different factors influencing the inclination to adopt fad diets. Specifically, this study explored the ways in which individual openness to following fad diets, participation in diet trends, and characteristics may influence attitudes towards fad diet adoption. The results of this study could provide knowledge on different factors that could influence the behavior of individuals regarding their intentions to adopt fad diets.

## 2. Conceptual Framework

### 2.1. Health Belief Model

According to LaMorte [26], health belief model (HBM) is highlighted as a prominent framework for understanding and predicting health-related behaviors. Within this model, several variables play crucial roles in shaping individuals’ decisions regarding health behaviors. These includes an individual’s perceived threat of sickness or disease (perceived susceptibility), belief of consequence (perceived severity), potential positive benefits of action (perceived benefits), perceived barriers to action, exposure to factors that prompt action (cues to action), and self-efficacy—a persons’ confidence in their ability to succeed [27]. It also emphasizes the importance of considering factors such as health motivation, people’s specific beliefs about susceptibility to health risks, and understanding the consequences of those risks [28]. However, despite its uses, the HBM has limitations, as it may not fully account for individual attitudes, habitual behaviors, social influences, or environmental or external factors that influence health choices.

Nutrition knowledge, encompassing an individual’s understanding of dietary guidelines and nutrient sources, is said to play a crucial role in shaping individuals’ dietary behaviors [29]. It serves as a determinant of diet-related behavior, influencing how individuals approach their eating habits [30]. Additionally, behavioral factors directly linked to nutrition knowledge are noteworthy, as studies indicate that individuals with specific dietary goals or practices tend to actively seek out nutrition information more than those without such goals [31,32]. This suggests that higher nutrition knowledge may influence individuals’ intentions regarding dietary choices, potentially impacting their inclination to adopt fad diets. Additionally, Spronk et al. [33] implicated that a lower level of nutrition knowledge is strongly associated with unhealthy eating habits, unbalanced dietary patterns, and an elevated risk of chronic diseases related to nutrition [34].

Furthermore, building upon the influence of knowledge about dietary behaviors, it is essential to recognize the pivotal role of health motivation in shaping individuals’ dietary choices and intentions. Previous research has highlighted a significant relationship between health motivation in food choices and positive dietary health behaviors [35,36,37]. Individuals who are highly motivated by a healthy lifestyle may demonstrate a greater willingness to explore novel dietary trends. This indicates that health motivation plays a pivotal role in shaping the intention to adopt fad diets. Moreover, motivation to enhance health is closely associated with the adoption of innovative dietary health promotion technologies, such as personalized nutrition [38,39,40,41]. These findings underscore the substantial impact of health motivation on individuals’ inclinations towards embracing fad diets, highlighting the importance of considering motivational factors in understanding dietary behavior.

Another factor affecting the adherence to dietary patterns is perceived barriers. In a study targeting older Australian adults adopting Mediterranean diets, they identified various perceived barriers to adherence. These include challenges related to the palatability of key foods, the insufficient availability of certain food items like red meat, and a lack of dietary variety [42]. Additionally, Zacharia et al. [43] identified factors such as the complexity of the dietary pattern, individual food preferences, perceived cost, and difficulties in purchasing, organizing, and preparing food as significant barriers. These findings highlighted the importance of recognizing and addressing perceived barriers to facilitate successful adoption of dietary patterns. Moreover, Pinho et al. [44], who focused on the barriers faced when it comes to eating healthy, determined that barriers such as lack of willpower, time constraints, and taste preferences strongly affected peoples’ dietary behavior.

Understanding the factors that influence individuals’ dietary behaviors involves not only recognizing perceived barriers but also acknowledging perceived benefits. Poinhos et al. [38] discovered a significant association between perceived benefits and the inclination to embrace personalized nutrition. Those who perceived greater benefits showed more positive attitudes and a stronger intention to adopt it. Additionally, higher nutrition self-efficacy was linked to favorable attitudes and a greater willingness to adopt personalized nutrition. Similarly, Mostafavi et al. [45] found that perceived benefits played a significant role in predicting students’ eating patterns, further supporting the importance of acknowledging perceived benefits in shaping dietary behaviors. Therefore, it was hypothesized that:

**H1:**  *The HBM domains have a significant effect on people’s intentions to adopt fad diets.*

### 2.2. Theory of Planned Behavior

The theory of planned behavior (TPB) is a widely recognized model for understanding behavior change, particularly in health contexts. It suggests that behavior is planned and driven by the strength of one’s intention to engage in it [46]. Evolving from the theory of reasoned action (TRA), the TPB introduced perceived behavioral control to account for instances where behavior may not be entirely voluntary [47]. Behavioral intention, central to the TPB, is influenced by attitudes towards behavior, subjective norms, and perceived behavioral control [48]. Individually, attitudes reflect one’s evaluation of behavior and its consequences, while subjective norms entail beliefs about others’ expectations. Moreover, perceived behavioral control involves beliefs about factors facilitating or hindering behavior. Overall, TPB provides a comprehensive framework for understanding the determinants of behavior change, highlighting the interplay between individual beliefs, social influences, and control over behavior.

An individual’s assessment of their interests can influence their resulting attitude and subsequent behavioral outcomes. This is said to ultimately affect intention [49]. Body image stands out as a key determinant, influencing the eating behaviors of adolescents with a notable inverse correlation reported between body satisfaction and engagement in dieting practices [50]. In an investigation done by Don et al. [51], perceived nutritional status (NS) served as a surrogate for body image, allowing for an assessment of middle-adolescent students’ contentment with their perceived NS. The findings revealed that adolescents commonly experience dissatisfaction with their bodies, even when their NS was within the normal range. This observation holds significance, given that existing studies indicated a positive association between heightened body dissatisfaction and the inclination to pursue weight loss strategies, including the adoption of weight loss and fad diets [52,53].

Apart from attitude, subjective norms can strongly influence an individual’s intention to adopt fad diets. This belief can be influenced by how much their social network and the media pressures them [54]. The interaction of overnutrition, media exposure, and peer influence creates a scenario where adolescents are vulnerable to adopting unhealthy dietary practices in their quest to achieve certain body goals [55]. In relation, studies suggest that as the degree of overnutrition intensifies, there is a corresponding increase in the propensity for adolescents to embark on dieting behaviors [56,57,58]. This underscores the intricate relationship between nutritional status, external influences, and the adoption of specific dietary habits among adolescents. Interestingly, in a study conducted by Don et al. [51] regarding the satisfactory level of students on different fad diets, the majority of the students’ satisfaction persisted despite documented instances of poor adherence to these diets and limited success in achieving weight loss. Consequently, their contentment or preference may be linked to the widespread popularity of fad diets, irrespective of their actual outcomes—thus, influenced by the public.

Similarly, perceived behavioral control also plays a significant role in determining an individual’s intention to adopt a fad diet. An individual possessing a sense of confidence in their ability to comply with the diet trend and perceives a degree of control over their actions tends to develop a favorable preference towards adopting it [59]. According to Mansourian et al. [60], intention includes thinking about performing the behavior and is the primary determinant of behavior. There is ample evidence that habits are another powerful predictor of eating behavior. Subsequently, when the power of habits increases, satisfactory experiences repeatedly reinforce behavior. The control of behavior among individuals adopting diet habits is therefore a common behavior. Additionally, Close et al. [54] revealed that the factors of beliefs, attitudes, subjective norms, and, notably, perceived behavioral control collectively elucidated a substantial portion of the variance in the intention to embrace a specific diet. This suggests that the perceived ability to navigate and adhere to specific fad diets plays a crucial and distinct role in shaping individuals’ intentions regarding their dietary choices. Thus, it can be hypothesized that:

**H2:**  *The TPB domains have a significant effect on people’s intentions to adopt fad diets.*

**Figure 1:**
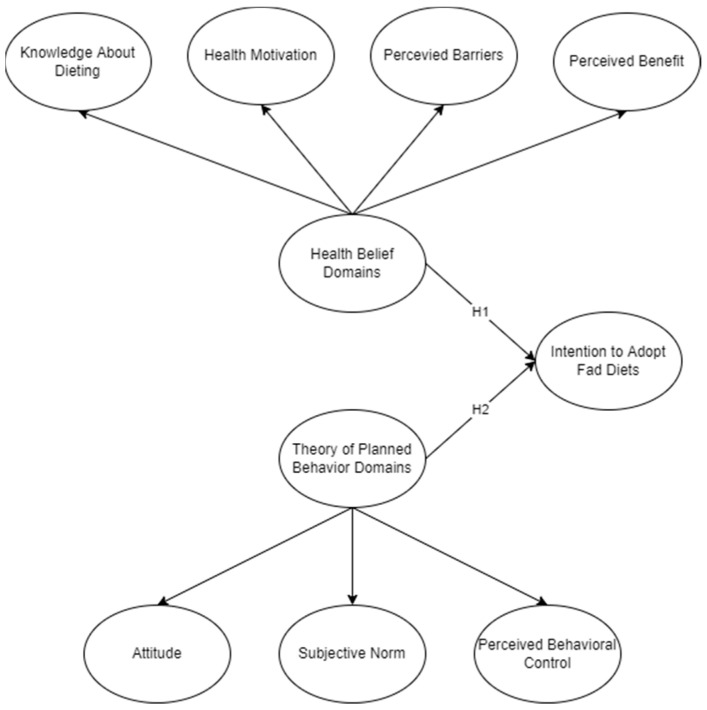
Conceptual framework.

The TPB and HBM are assessed simultaneously as presented in the conceptual framework (Figure 1). The TPB is well-known for its capacity to clarify specific actions by investigating attitudes, subjective norms, and perceived behavioral control [61]. According to recent studies, the TPB as a sole framework might not fully capture all decisions pertaining to health or overall behavior outside of the theory domains [46]. The usual solution to cover the gap or to improve the behavioral findings is to consider adding more components, variables, or constructs to the TPB [62,63,64]. On the other hand, the HBM provides a more thorough analysis of health-related attitudes and actions [65]. This has been used to assess perception and belief among individuals when dealing with studies related to health [27,28,29]. As a reflection of their theoretical distinctions, this study assessed the adoption of fad diets—whether due to behavioral aspects or health perception—through the use of a higher-order reflective construct of the TPB and HBM. A total of two hypotheses were developed to facilitate this comparative analysis.

## 3. Methodology

### 3.1. Participants

The demographic profiles and dietary habits of the respondents were gathered through a comprehensive questionnaire survey, distributed via Google Forms. The data collection process spanned from November 2023 until January 2024. Prior to distribution, this study was approved by the Mapua University Ethics Committee (FM-RC-23-01-75), signed by the research coordinator, Dr. Ma. Janice J. Gumasing, and the dean, Dr. Michael Young, last 29 October 2023. The survey reached a total of 407 participants through a multifaceted dissemination approach, utilizing social media platforms (particularly group pages dedicated to discussions on fad diets), peer-to-peer sharing, and face-to-face surveying facilitated through QR code scans.

To ensure the reliability of the findings and their representativeness of the Filipino population, the sample size of 400 was determined using the Yamane Taro [66] formula. This sample size is consistent with a strong dataset, given a total Filipino population of 113,000,000 and a margin of error 0.05. The respondents’ demographic traits and dietary preferences provided valuable information that can reflect the general Filipino population. As a reflection of the sample size, the Philippine Statistics Authority (PSA) [67] determined that the percent distribution of women (49.4%) younger than 64 years old comprised 89.87% of the population. The current study targeted as representative a sample as possible using a purposive sampling technique. In addition, the median range of the population is 25 years old. These categories were chosen because the generalizability of the practitioners of fad diets is focused on these characteristics. Brehm et al. [7] and Whyte et al. [8] explained that mostly women and younger generations are practicing different fad diets.

Table 1 provides an overview of the respondents’ demographic profiles in various categories. The data were collected in a cross-sectional format via a purposive sampling method, and only those who were knowledgeable regarding fad diets or had been practicing different fad diets were considered as valid respondents. The data showed that 47.4% of the respondents identified as male, while the remaining 52.6% identified as female. In terms of age distribution, participants were segmented into the following categories: 74.2% between 18 and 24 years old, 16.7% in the 25–34 age range, 6.6% in the 35–44 age range, 2.2% in the 45–54 age range, and the remaining 0.2% were 55 years old and older. Concerning living environments, 47.7% of respondents resided in rural areas, while urban settings were where the majority of participants, accounting for 52.3%, resided. The participants’ occupational status was classified as follows: 73% were students, 20.9% were employees or self-employed, 4.4% were employers or business owners, and the remaining 1.7% were unemployed. In addition, the monthly income or allowance distribution revealed that 34.4% of respondents earned less than 5000 PHP, 25.8% earned between 5001–10,000 PHP, 19.4% earned 10,001–20,000 PHP, 11.8% earned 20,001–30,000 PHP, and the rest, comprising 8.6%, earned 30,001 PHP or higher.

The survey also explored participants’ engagement with fad diets, with 69.5% confirming that they had tried following a fad diet. Among the popular fad diets, 51.6% had experimented with intermittent fasting, 17% with a vegan diet, 32.4% with a low-fat diet, 31.4% with a low-carb diet, 11.1% with the keto Diet, and 17.7% with juice cleansing. Lastly, 8.1% had tried other fad diets, leaving a blank for them to specify. Respondents discovered these fad diets through various channels, with 46.4% citing social media as their source, 28.3% through friends or family, 11.3% through articles or blogs, 10.6% through health professionals, and 3.4% through books or magazines.

### 3.2. Measured Items

The questionnaire was designed to identify the significant factors influencing individuals’ intentions to adopt various fad diets. The demographics section aimed to gather diverse information from participants, including sex, age, educational attainment, occupational status, monthly allowance or income, types of fad diets previously tried, frequency of engagement in dieting, and whether they had attempted adopting a fad diet before.

The reflective higher-ordered HBM and TPB framework included 4 and 3 variables, respectively. Participants expressed their responses on a five-point scale ranging from strongly disagree (1) to strongly agree (5). The questionnaire’s variables were abbreviated as (1) knowledge about dieting (KD), (2) perceived benefit (PB), (3) health motivation (HM), (4) perceived barriers (BAR), (5) attitude (AT), (6) subjective norm (SN), (7) perceived behavioral control (PBC), and (8) intention to adopt fad diets (INT). Presented in Table 2 are the adapted measured items.

Prior to full deployment, a pilot test was conducted to check for grammatical errors, understanding, and overall reliability. There were only a few grammatical errors that needed to be changed, and the overall Cronabch’s alpha score was determined to be 0.891—deemed to be acceptable [79]. Moreover, a test for normality was conducted to verify that the collected data were applicable for analysis. It was seen that the outputs using the Shapiro–Wilk test were all within threshold the set by Hair [80], ±1.96. In addition, the common bias method to fully utilize the dataset was performed using Harman’s single-factor test. As suggested by Podsakoff et al. [81], the output should have a total variance less than 50%. The collected sample only achieved an overall of 19.856%.

### 3.3. Structural Equation Modeling

Structural equation modeling (SEM) is utilized for the analytical measurement of relationships among latent and observed variables through a set of statistical techniques [66]. As per Kaplan [82], SEM can be characterized as a class of techniques that tries to address speculations about the means, differences, and variables of observed information in terms of fewer ‘primary’ boundaries characterized by a hidden estimated or calculated hypothetical model. As per Xiao [83], SEM is a general way to deal with analysis among observed and unobserved variables [84]. Objective or reflective constructs could be holistically assessed using measured items from established models that considered similar theories for establishing the inter-relationship and causal relationship identification [80]. In relation, Fan et al. [85] also expounded on its applicability not only in ecological-related studies, but also among consumer behavior studies.

Different SEM tools can be considered, but the research conducted by Dash and Paul [79] highlighted the numerous advantages of using partial least squares SEM (PLS-SEM), including its flexibility in establishing the measurement model through composites, accommodating both reflective and formative models, developing new measures, and offering more configuration options compared to alternative methods. Moreover, the study of Sarstedt et al. [86] explained that one limitation of conducting SEM is the presence of several mediating variables. This leads to multiple path analyses and raises the error of calculation. A higher-order reflective construct may be employed as long as the domains are grounded by established theories [87]. Hence, the current study utilized this approach to examine the latent variables influencing health beliefs and conduct a behavioral analysis of fad diets using SMART-PLS v3.0 software.

## 4. Results and Discussion

**Figure 2:**
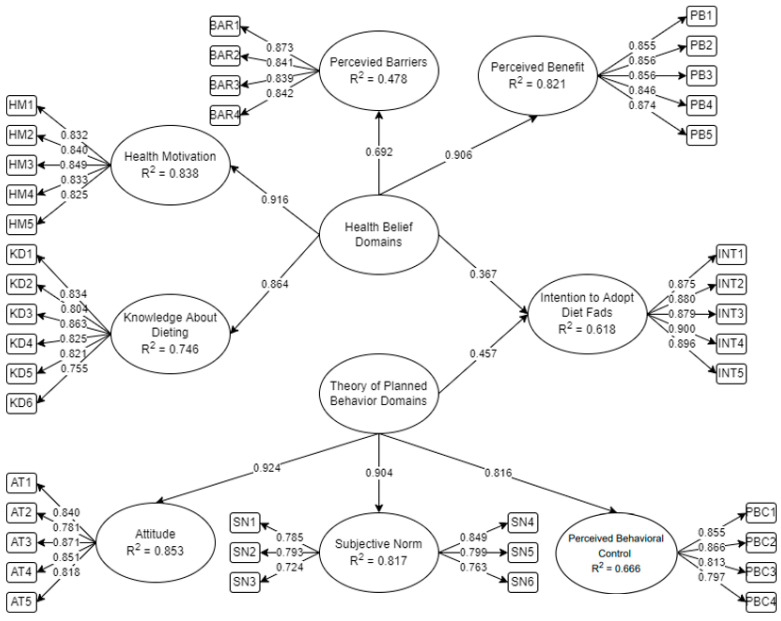
Structural equation modeling.

The SEM for determining the factors influencing individuals’ intention to adopt various fad diets is presented in Figure 2. The relationships among higher-ordered constructs of both the HBM and TPB exhibited a significant output (*p*-value < 0.05) [83]. Moreover, all the measured items were deemed significant (factor loading > 0.70) [74]. Therefore, the output represents the final model for assessing factors affecting intention to adopt dieting fads. The descriptive statistics of the factors are presented in Table 3.

It could be seen from the output that the behavioral domains of the TPB presented the highest effect on the intention to adopt fad diets (β: 0.457, *p* < 0.001). Among these, people’s attitudes had the most influence (β: 0.924, *p* < 0.001), followed by subjective norms (β: 0.904, *p* < 0.001), and perceived behavioral control as the least (β: 0.816, *p* < 0.001).

Defined as the individual preferences or assessments of the perceived good or adverse impacts of engaging in that practice, attitude was the most influential domain. The study of Aggrawal et al. [88] examined the relationships between preferences and attitudes about health when consuming specific foods or nutrients. The results showed that people with positive attitudes about health are more likely to choose a healthier diet than those with negative attitudes. In relation to this study, the respondents reflected that fad diets are pleasing, satisfying, enjoyable, interesting, and useful in their daily lives, and they allowed them to be healthy (perception). Evidenced by the study of Vaughan et al. [89], people have different attitudes towards a certain behavior. It was presented that if people either have negative attitude towards exercising and have a moderate diet or have fewer barriers and benefits, then greater engagement with a certain lifestyle is seen. In relation, people find adopting fad diets to be behaviorally easier compared to going to fitness centers or exercising.

Supported by the study of Ong et al. [90], Filipinos only have limited time to engage in fitness and exercising. This is because of daily tasks and activities needed to be accomplished, which take a toll (e.g., travel time is longer due to traffic, expensive centers, overall precursor to engage in the activity). This study supports how perceived behavioral control was the least significant output. From the indicators, people believed that they had the discipline to follow a strict diet, remain motivated, have enough resources (i.e., time and money), and have the overall control to follow fad diets. As reflected, numerous fad diets claim to offer quick and specific health benefits, which creates a perception among consumers that they will achieve immediate results [78]. However, according to Chua et al. [91], while consumers may experience short-term benefits from such fads, they may face difficulties sustaining control over their dietary choices over an extended period; therefore, it could be posited that sustaining it would not be highly plausible among most individuals.

Hardin-Fanning and Ricks [92] elaborated that it is not solely an individuals’ own control that greatly influences eating habits, but their environment as well. This claim supports why subjective norms had higher effects than perceived behavioral control. From this study, people influencing an individual does not really affect the adoption of fad diets. In accordance, support from loved ones, acceptance, and overall commitment are bolstered up. From the timeframe study by Horne et al. [93], it was seen that the more health-conscious the group an individual belongs to, the more they are inclined to adopt the same lifestyle, which could be related to this study by means of social support. As no negative connotation is evident, people would continuously adopt the fad diet. However, the need for proper professional input should still be considered and should not depend only on social groups. This is why health belief (HBM domains) significantly affected people’s intentions to adopt fad diets (β: 0.367, *p* < 0.001).

Supporting the constructs, the results presented in Table 4 indicate that all items considered within the model met the required standards for internal validity and reliability. The Cronbach’s alpha (CA) ranged from 0.853 to 0.949, and composite reliability (CR) ranged from 0.901 to 0.954. The values for the constructs both exceeded 0.70, which indicates that convergent validity was achieved [79,83]. Furthermore, the average variance extracted (AVE) had a range of 0.515 to 0.785 for each construct—surpassing the 0.50 threshold. This means that the measured items characterized the latent variables.

In variance-based SEM, such as partial least squares, the effectiveness of a model can be assessed using the Fornell–Larcker criterion (Table 5) and cross-loading investigation [85]. A heterotrait–monotrait ratio (Table 6) below 0.85 across reflective constructs validates the discriminant validity. In accordance, the Fornell–Larcker criterion should have higher diagonal constructs compared to the valued correlation for discriminant validity to be supported [94]. As all thresholds were achieved, the general findings are accepted across all constructs. This indicated that the overall representation among the relationships made are significant and that reflective ordered constructs are deemed to appropriately represent the domains of the TPB and HBM.

In this case, it could be posited that despite differences in significance output, health beliefs would also still measure an individual’s intention to adopt fad diets. As reflected, health motivation had the highest significance (β: 0.916, *p* < 0.001), followed by perceived benefit (β: 0.906, *p* < 0.001), knowledge about dieting (β: 0.884, *p* < 0.001), and finally, perceived barriers (β: 0.692, *p* < 0.001). Highlighting health motivation, people believed that following fad diets allow them to have a healthier lifestyle and reduce weight-related diseases (e.g., manage blood sugar levels, cholesterol, and supports heart health), and are impactful for their overall well-being. As reflected by Bali and Naik [14], it really does reduce certain coronary and weight problems. However, this was only found among vegan and vegetarian diets.

It was also seen that people believed following fad diets promotes quick and easy weight loss, as well as providing better mood and overall mental health. The better mood and overall mental health connotation, however, has been proven not to be true [14]. As discussed, studies have shown that following fad diets is not recommended by health experts and could lead to health complications [20,21]—especially when micronutrient and caloric deficits are being practiced but are not advised. However, the results could reflect an implication of the lack of knowledge among Filipinos on fad diets. To which, Filipinos could still be encouraged to adopt these diet practices.

From the indicators, it was presented that people believed that following fad diets improves physical appearances, helps in practicing discipline, boosts energy for everyday activities, and even boosts self-esteem. Though proven to really boost self-esteem [7,8], the health benefit perception highlights the lack of understanding among individuals adopting fad diets. This is contrary since knowledge about dieting was seen to be a significant variable. In accordance, people indicated that they posses the knowledge and understanding of the different fad diets, their health benefits, and the risks associated with them. People expressed that they know that fad diets are applicable to certain people, and could not be generalized overall. This is interesting since Don et al. [51] provided evidence of the reason why younger Filipinos adopt fad diets. It was evaluated that most practices are due to low self-esteem and body satisfaction, and promoting the practice due to quick results rather than health.

This means that intervention, proper knowledge dissemination, and assessment should be performed. In addition, perceived barriers were the least significant variable reflecting the HBM. Supported by similar studies [91,95], people indicated that this was difficult to sustain, especially the monitoring of caloric values, controlling cravings, and devoting more time and effort to the fad diet. However, the significant factors of the intention to adopt fad diets indicated that they still see themselves adopting fad diets, want to do it in the future, and hope for sustainable adoption. Reflecting on Steinhauser et al. [96], they showed that likelihood or intention increases with perceived healthiness. If one considers the factors that impact the credibility of a product’s health advantages, all of this also becomes a factor that raises the willingness among individuals. Moreover, Kim [95] found that individuals can develop coping mechanisms or alternative strategies to manage perceived barriers effectively, thereby reducing the impact of these barriers on their attitudes. Hence, the results indicate that even if individuals perceive challenges in adopting a particular diet trend, it does not significantly affect their overall attitude towards it. This raises a concern regarding fad diet adoption that governing bodies and health professionals should focus on.

### Model Fit Analysis

The model fit analysis was conducted to evaluate the validity of the model. Table 7 demonstrates that all parameter estimates exceeded the threshold requirements for all measures such as the standardized root mean residual (SRMR), Chi-squared, and normal fit index (NFI). This indicates that the suggested model is acceptable. Following the suggestion of Dash and Paul [68], the usual model fit of PLS-SEM only includes the three parameters while being supported by other measurements like discriminant and convergent validity. Following the suggested minimum cut-off, it could be posited that the model is deemed to be acceptable. Summarized in Table 8 are the hypothesis decisions, beta coefficients, and *p*-values.

## 5. Conclusions

The structural equation modeling (SEM) analysis conducted in this study has provided valuable insights into the factors influencing individuals’ intention to adopt various fad diets among Filipinos. The SEM results revealed significant relationships among the higher-ordered constructs of both the health belief model (HBM) and the theory of planned behavior (TPB), with all measured items demonstrating a significant output. Notably, the findings indicated that behavioral domains from the TPB had the strongest effect on the intention to adopt fad diets, with attitude emerging as the most influential domain, followed by subjective norms and perceived behavioral control. This suggests that Filipinos’ attitudes towards fad diets, influenced by perceptions of their health benefits and social pressures, play a pivotal role in shaping their intention to adopt such diets.

Furthermore, the study underscored the significant impact of health motivation, perceived benefit, knowledge about dieting, and perceived barriers—components of the HBM—on individuals’ intention to adopt fad diets. Health motivation emerged as the most influential factor, highlighting the importance of individuals’ desire for a healthier lifestyle and their perception of fad diets as a means to achieve it. However, it is important to note that while individuals may perceive fad diets as offering quick and easy weight loss and other benefits, evidence suggests that many of these diets lack adaptability, sustainability, and may pose health risks. Thus, interventions aimed at disseminating accurate information and promoting healthy dietary practices are crucial.

As a reflection on the findings of this study, they contribute to the existing literature by offering insights into the complex interplay of factors influencing fad diet adoption. By understanding these factors, policymakers, health professionals, and educators can develop targeted interventions to promote informed dietary choices and improve public health outcomes.

### 5.1. Theoretical Implications

Dietary fads are one of the most dynamic and popular aspects of health behavior due to the widespread occurrence of obesity. On a global scale, dietary fads have led to a boom in public awareness regarding dieting, with people increasingly resorting to fad diets as alleged remedies for their persistent health issues [1]. This research carefully examined the complex areas of fad diets, highlighting the many variables affecting people’s intentions to follow these dietary patterns. Building on a strong theoretical base, a higher-ordered reflective construct of the TPB for behavioral analyses and the HBM for health beliefs was considered. Evident from the results, it was seen that the measured items were all deemed significant as well as the variables. In addition, all were deemed to be valid and reliable. This reflects that the construct and measured items could be adapted and expanded for other health-related studies.

This study was also able to provide evidence supporting the higher-order reflective construct suggested by Sarstedt et al. [86]. It was appropriate to use this model for comparison, as well as highlighting the intricate interactions between social, psychological, and health-related variables. According to Gerend and Shepherd [100], both theories highlight the significance of many types of beliefs in predicting an individual’s behavior since it contains overlapping elements. Consequently, it is imperative to integrate the two theories in order to pinpoint certain constructs that impact particular behaviors, thereby enhancing our comprehension of risk prevention behaviors.

Our findings provide an entirely new perspective on theoretical frameworks in addition to providing significant insights into the factors influencing the acceptance of fad diets. Through a combined evaluation of the TPB and HBM, we addressed the drawbacks of single-framework implementations. Encompassing both models allowed us to leverage the strengths of each framework. This is why the dual approach offered a better understanding of health behaviors, overcoming individual model limitations and enhancing predictive capacity. There are many variables that influence dietary decisions; therefore, using a single framework may not adequately explain the complex reasons people adopt or reject particular dietary trends. With the combination of attitudes, subjective norms, perceived behavioral control, and health beliefs, a more thorough picture of health behavior is revealed. Interestingly, our research demonstrated the beneficial effects of merging these frameworks, demonstrating how the integration of these frameworks improves health behavior monitoring in relation to fad diets. Our findings support a wider use of comprehensive theoretical methods in the study of complex health issues, while also advancing our understanding of health behaviors connected with dietary trends.

### 5.2. Practical and Managerial Implications

#### 5.2.1. For Government Agencies

This study’s comprehensive examination of the factors influencing people’s intentions to adopt various fad diets offers useful information to government agencies tasked with tackling public health concerns. The results emphasized the need for targeted, evidence-based health communication strategies. Using this information, governmental entities may develop campaigns that highlight the risks associated with adopting particular fad diets—particularly those with little evidence to back it up. The study also emphasizes how people’s intentions about fad diet adoption are shaped by their knowledge, perceived benefits, health motivation, perceived barriers, attitude, subjective norms, and perceived behavioral control. Comprehending these components enables customized approaches that address certain variables influencing individuals’ decisions to adhere to fad diets. To which, governmental organizations may design more effective educational initiatives and rules to promote the uptake of nutritious eating practices and decrease the creation of potentially harmful fad diets.

#### 5.2.2. For Health Practitioners

The findings of this study provide helpful guidance for those who are considering adopting fad diets to make educated dietary decisions. The study clarifies important variables, that together, affect people’s intentions to follow fad diets, including knowledge about dieting, perceived benefits, health motivation, perceived barriers, attitude, subjective norm, and perceived behavioral control which collectively influenced the intention to adopt fad diets. With the use of this information, people are better equipped to assess food trends objectively, considering their risks, potential health advantages, and scientific foundation. It could be posited that proper guidance, though being conducted, should be highlighted among adopters of fad diets. People may make decisions that support their health objectives and steer clear of the risky fad diets by being aware of these aspects. To which, health practitioners may try to embed the necessary information with regards to the intended health goals among adopters. Highlighting the benefits and consequences may reduce bad recommendations among non-health practitioners and clinicians. The study highlights how crucial it is to look for trustworthy information, speak with medical specialists, and be conscious of any barriers or motivating factors. Ultimately, individuals can use the knowledge obtained to adopt sustainable, evidence-based dietary practices that contribute to long-term health and well-being.

### 5.3. Limitations and Future Research

Our study, anchored in survey methodology, offers valuable insights into fad diet adoption. However, its reliance solely on surveys imposes limitations on the depth of understanding participants’ behaviors. To address this, future researchers can embrace mixed-method approaches, pairing surveys with qualitative interviews or focus group discussions. This combination would unveil a more nuanced comprehension of individuals’ motivations and experiences with fad diets, providing a richer dataset for analysis.

Moving forward, researchers could benefit from diversifying analytical tools beyond structural equation modeling (SEM). While SEM offers robust insights, integrating machine learning techniques or data mining can unveil hidden patterns and relationships that traditional SEM might overlook. Additionally, supplementing quantitative findings with a qualitative analysis, such as a thematic analysis or grounded theory, would provide a deeper exploration of participants’ beliefs and attitudes, contributing to a more comprehensive understanding of the complex landscape of fad diet adoption. As reflected by the study of Razzaq et al. [101], it could be posited that self-reported bias may be present since a judgement of the agreeable statement under the constructs is made. The adoption of the measured items may be validated, but future researchers may employ a qualitative–quantitative approach for a holistic conclusion and report build-up.

Furthermore, transitioning from a snapshot approach to longitudinal studies would capture the dynamic evolution of attitudes and behaviors over time, shedding light on the sustainability of fad diet adoption. Since this study only employed convenience sampling by filtering questions on fad diet consumers, it can still be posited that not all insights may have been captured for generalization. In accordance, the sample size, characteristics, and overall behavior may still not be representative of a larger perspective. As a reflection, this study can only be generalized among the younger generation and young adult women who have experienced or are adopting fad diets; thus, not the whole Filipino population.

## Figures and Tables

**Table 1:** Demographic Profile.

Respondent Profile	Category	N	%
Sex	Male	193	47.4%
	Female	214	52.6%
Age	18–24 years old	302	74.2%
	25–34 years old	68	16.7%
	35–44 years old	27	6.6%
	45–54 years old	9	2.2%
	55 years old and older	1	0.2%
Living Environment	Rural	194	47.7%
	Urban	213	52.3%
Occupational Status	Student	297	73%
	Employee/Self-employed	85	20.9%
	Employer/Business Owner	18	4.4%
	Unemployed	7	1.7%
Monthly Allowance/Income	Less than 5000 PHP	140	34.4%
	5001–10,000 PHP	105	25.8%
	10,001–20,000 PHP	79	19.4%
	20,001–30,000 PHP	48	11.8%
	30,001 or Higher	35	8.6%
Have you ever tried to follow a diet fad	Yes	283	69.5%
	No	124	30.5%
Which among the fad diets have you tried?	Intermittent Fasting	210	51.6%
	Vegan Diet	69	17%
	Low-fat Diet	132	32.4%
	Low-carb Diet	128	31.4%
	Keto Diet	45	11.1%
	Juice Cleansing	72	17.7%
	Others:	35	8.1%
How did you find out about this diet fad?	Social Media	189	46.4%
	Friends or Family	115	28.3%
	Articles/Blogs	46	11.3%
	Health Professionals	43	10.6%
	Books/Magazines	14	3.4%

**Table 2:** Measured Items.

Variable	Code	Description	References
Knowledge about Dieting	KN1	I possess knowledge about different dieting trends	Ratilla et al. [68]
	KN2	I have a good understanding of the effects linked to different diet fads	Ratilla et al. [68]
	KN3	I have a good understanding of the health benefits of various diets	Ratilla et al. [68]
	KN4	I am familiar with the potential benefits and risks associated with trendy diets	Ratilla et al. [68]
	KN5	I already have an idea about how to start implementing diet fads	Ratilla et al. [68]
	KN6	I am aware that diet trends may work differently for other people	Ratilla et al. [68]
Perceived Benefit	PB1	I think that diet fads can improve my overall appearance especially in terms of skin	Tahreem et al., [1]
	PB2	I think following diet fads brings a heightened sense of discipline and structure to my eating habits	Fey, Tina., [69]
	PB3	I think diet fads emphasize nutrient-dense foods which can boosts energy and focus	Cohen, M., [70]
	PB4	I think following diet fads can increase my self esteem	Zychowicz et al. [71]
	PB5	I think following diet fads can help increase my knowledge about food nutrition	Zychowicz et al. [71]
Health Motivation	HM1	I am motivated with the goal of improving my overall health and well-being through targeted nutritional choices	Brennan et al., [72]
	HM2	I believe that it addresses specific health concerns, such as reducing cholesterol levels, managing blood sugar, or supporting heart health	Hanson, B., [73]
	HM3	It would support my mental health, believing that certain nutritional approaches can positively impact mood, cognition, and overall mental well-being	Diet and mental health., [74]
	HM4	I believe that following diet fads can be a starter towards a healthy lifestyle	Zychowicz et al. [71]
	HM5	I believe that following diet fads can provide a quick and effective way to lose weight	Zychowicz et al. [71]
Perceived Barriers	BAR1	I find it challenging to sustain motivation for diet fads due to the often-restrictive nature in terms of diet	Lichtenstein et al., [75]
	BAR2	I find it difficult to pay attention to caloric values of food I eat	Zychowicz et al. [71]
	BAR3	I find it difficult to keep myself from craving food restricted by my diet	Bech-Larsen et al. [76]
	BAR4	I find it difficult to give effort and time in paying attention to diet fads	Bech-Larsen et al. [76]
Attitude	AT1	Adopting a diet fad is pleasing	Bech-Larsen et al. [76]
	AT2	Adopting a diet fad will allow me to be healthy	Bech-Larsen et al. [76]
	AT3	Adopting a diet fad can be satisfying for me	Bech-Larsen et al. [76]
	AT4	Adopting a diet fad can be useful in my life	Bech-Larsen et al. [76]
	AT5	I find adopting a diet fad enjoyable and interesting	Bech-Larsen et al. [76]
Subjective Norm	S1	My family does not mind me following different diet fads for weight loss	Verheijden et al. [77]
	S2	My friends will support my decision in following diet fads	Verheijden et al. [77]
	S3	In social gatherings, people are not displeased if I refuse food offers	Verheijden et al. [77]
	S4	I feel like my peers or colleagues will understand my commitment for diet fads	Verheijden et al. [77]
	S5	Most people I look up to has the intention to adopt a diet fad	Biasini et al. [78]
	S6	Most people I look up to is accepting of diet fads	Biasini et al. [78]
Perceived Behavioral Control	PBC 1	I have enough discipline to follow a strict diet fad	Biasini et al. [78]
	PBC2	I am motivated enough to follow a strict diet fad	Biasini et al. [78]
	PBC3	I have enough time and money to plan out a strict a diet fad	Biasini et al. [78]
	PBC4	If I wanted to, I could follow a diet fad with many food restrictions	Biasini et al. [78]
Intention to Adopt Diet Fads	INT1	I predict myself to adopt a diet fad in the future	Biasini et al. [78]
	INT2	I want to adopt a diet fad in the future	Biasini et al. [78]
	INT3	I will adopt a specific diet fad in the future	Biasini et al. [78]
	INT4	I plan on adopting a specific diet fad in the future	Biasini et al. [78]
	INT5	I am hoping to adopt a diet fad in the future	Biasini et al. [78]

**Table 3:** Statistical Analysis of Indicators.

Variable	Item	Mean	Standard Deviation	Factor Loading
Knowledge about Dieting	KD1	3.471	1.141	0.834
	KD2	3.587	1.104	0.804
	KD3	3.683	1.163	0.863
	KD4	3.741	1.088	0.825
	KD5	3.409	1.191	0.821
	KD6	4.062	1.132	0.755
Perceived Benefit	PB1	3.574	1.186	0.855
	PB2	3.868	1.117	0.856
	PB3	3.736	1.117	0.856
	PB4	3.781	1.074	0.846
	PB5	3.878	1.142	0.874
Health Motivation	HM1	3.893	1.104	0.832
	HM2	4.005	1.021	0.840
	HM3	3.868	1.150	0.849
	HM4	3.958	1.062	0.833
	HM5	3.808	1.128	0.825
Perceived Barrier	BAR1	3.579	1.179	0.873
	BAR2	3.576	1.190	0.841
	BAR3	3.559	1.138	0.839
	BAR4	3.491	1.169	0.842
Attitude	AT1	3.726	1.156	0.840
	AT2	3.850	1.081	0.781
	AT3	3.586	1.142	0.871
	AT4	3.796	1.142	0.851
	AT5	3.459	1.181	0.818
Subjective Norms	SN1	3.606	1.171	0.785
	SN2	3.666	1.116	0.793
	SN3	3.389	1.145	0.724
	SN4	3.681	1.079	0.849
	SN5	3.446	1.140	0.799
	SN6	3.618	1.117	0.763
Perceived Behavioral Control	PBC1	3.369	1.136	0.855
	PBC2	3.449	1.133	0.866
	PBC3	3.242	1.188	0.813
	PBC4	3.514	1.199	0.797
Intention to Adopt Fad Diets	INT1	3.651	1.133	0.875
	INT2	3.741	1.118	0.880
	INT3	3.810	1.094	0.879
	INT4	3.803	1.132	0.900
	INT5	3.840	1.154	0.896

**Table 4:** Convergent Validity.

Variable	Code	CA	CR	AVE
HBM Domains	HBM	0.949	0.954	0.515
Attitude	AT	0.889	0.919	0.693
Perceived Barrier	BAR	0.871	0.912	0.721
Intention to Adopt Fad Diets	INT	0.932	0.948	0.785
Knowledge about Dieting	KD	0.901	0.924	0.669
Perceived Benefit	PB	0.910	0.933	0.735
Perceived Behavioral Control	PBC	0.853	0.901	0.695
Subjective Norms	SN	0.876	0.907	0.618
TPB Domains	TPB	0.934	0.942	0.522

**Table 5:** Fornell–Larcker Criterion.

	AT	BAR	HBM	HM	INT	KD	PB	PBC	SN	TPB
AT	0.833									
BAR	0.450	0.849								
HBM	0.685	0.692	0.717							
HM	0.770	0.550	0.616	0.836						
INT	0.702	0.486	0.714	0.671	0.886					
KD	0.658	0.466	0.664	0.704	0.664	0.818				
PB	0.739	0.521	0.706	0.815	0.665	0.685	0.858			
PBC	0.667	0.354	0.584	0.527	0.605	0.598	0.471	0.833		
SN	0.752	0.503	0.658	0.719	0.687	0.647	0.683	0.588	0.786	
TPB	0.624	0.501	0.614	0.712	0.706	0.719	0.623	0.716	0.704	0.722

**Table 6:** Heterotrait–Monotrait (HTMT) Ratio.

	AT	BAR	HBM	HM	INT	KD	PB	PBC	SN
AT									
BAR	0.512								
HBM	0.849	0.783							
HM	0.835	0.623	0.786						
INT	0.770	0.537	0.784	0.734					
KD	0.734	0.519	0.831	0.778	0.721				
PB	0.823	0.583	0.764	0.803	0.720	0.750			
PBC	0.762	0.407	0.647	0.598	0.674	0.683	0.530		
SN	0.832	0.574	0.828	0.812	0.757	0.724	0.763	0.678	
TPB	0.806	0.554	0.839	0.843	0.807	0.782	0.787	0.818	0.801

**Table 7:** Model Fit.

Measures	Parameter Estimates	Minimum Cut-Off	References
SRMR	0.059	<0.08	Hu & Bentler [97]
Chi-squared	2.694	<5.00	Hooper [98]
NFI	0.843	>0.80	Baumgartner [99]

**Table 8:** Summarized Hypothesis Test.

Hypotheses	Relationship	Beta Coefficient	*p*-Value	Decision
1	HBM → INT	0.367	<0.001	Accept
2	TPB → INT	0.457	<0.001	Accept
Higher-Order	HBM → KD	0.884	<0.001	Accept
Higher-Order	HBM → HM	0.916	<0.001	Accept
Higher-Order	HBM → BAR	0.692	<0.001	Accept
Higher-Order	HBM → PB	0.906	<0.001	Accept
Higher-Order	TPB → AT	0.924	<0.001	Accept
Higher-Order	TPB → SN	0.904	<0.001	Accept
Higher-Order	TPB → PBC	0.816	<0.001	Accept

## Data Availability

The data presented in this study are available on request from the corresponding author. The data are not publicly available due to privacy restrictions.
